# Identifying new lignin bioengineering targets: 1. Monolignol-substitute impacts on lignin formation and cell wall fermentability

**DOI:** 10.1186/1471-2229-10-114

**Published:** 2010-06-17

**Authors:** John H Grabber, Paul F Schatz, Hoon Kim, Fachuang Lu, John Ralph

**Affiliations:** 1 U.S. Dairy Forage Research Center, USDA-Agricultural Research Service, Madison, Wisconsin 53706, USA; 2 Department of Biochemistry and DOE Great Lakes Bioenergy Research Center, University of Wisconsin, Madison, Wisconsin, USA

## Abstract

**Background:**

Recent discoveries highlighting the metabolic malleability of plant lignification indicate that lignin can be engineered to dramatically alter its composition and properties. Current plant biotechnology efforts are primarily aimed at manipulating the biosynthesis of normal monolignols, but in the future apoplastic targeting of phenolics from other metabolic pathways may provide new approaches for designing lignins that are less inhibitory toward the enzymatic hydrolysis of structural polysaccharides, both with and without biomass pretreatment. To identify promising new avenues for lignin bioengineering, we artificially lignified cell walls from maize cell suspensions with various combinations of normal monolignols (coniferyl and sinapyl alcohols) plus a variety of phenolic monolignol substitutes. Cell walls were then incubated in vitro with anaerobic rumen microflora to assess the potential impact of lignin modifications on the enzymatic degradability of fibrous crops used for ruminant livestock or biofuel production.

**Results:**

In the absence of anatomical constraints to digestion, lignification with normal monolignols hindered both the rate and extent of cell wall hydrolysis by rumen microflora. Inclusion of methyl caffeate, caffeoylquinic acid, or feruloylquinic acid with monolignols considerably depressed lignin formation and strikingly improved the degradability of cell walls. In contrast, dihydroconiferyl alcohol, guaiacyl glycerol, epicatechin, epigallocatechin, and epigallocatechin gallate readily formed copolymer-lignins with normal monolignols; cell wall degradability was moderately enhanced by greater hydroxylation or 1,2,3-triol functionality. Mono- or diferuloyl esters with various aliphatic or polyol groups readily copolymerized with monolignols, but in some cases they accelerated inactivation of wall-bound peroxidase and reduced lignification; cell wall degradability was influenced by lignin content and the degree of ester group hydroxylation.

**Conclusion:**

Overall, monolignol substitutes improved the inherent degradability of non-pretreated cell walls by restricting lignification or possibly by reducing lignin hydrophobicity or cross-linking to structural polysaccharides. Furthermore some monolignol substitutes, chiefly readily cleaved bi-phenolic conjugates like epigallocatechin gallate or diferuloyl polyol esters, are expected to greatly boost the enzymatic degradability of cell walls following chemical pretreatment. In ongoing work, we are characterizing the enzymatic saccharification of intact and chemically pretreated cell walls lignified by these and other monolignol substitutes to identify promising genetic engineering targets for improving plant fiber utilization.

## Background

**Figure 1:**
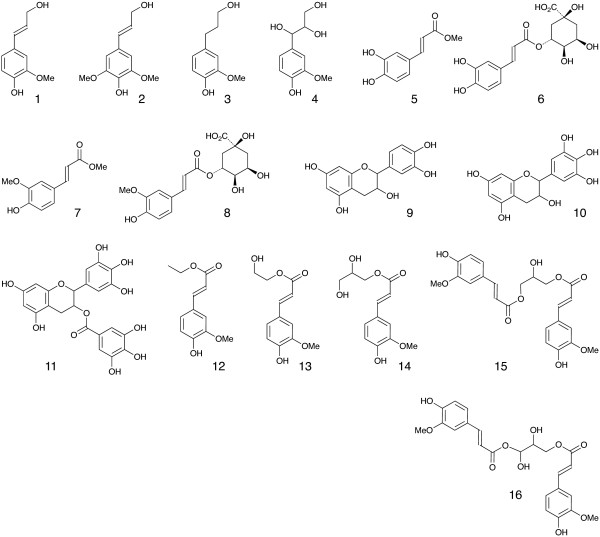
**Monolignols and monolignol substitutes used to artificially lignify maize cell walls**. Coniferyl alcohol **1 **and sinapyl alcohol **2 **are the primary monolignols used by angiosperms to form lignin. In our first experiment, we examined partial substitution of **1 **and **2 **with dihydroconiferyl alcohol **3**, guaiacylglycerol **4**, methyl caffeate **5**, caffeoylquinic acid **6**, methyl ferulate **7**, feruloylquinic acid **8**, epicatechin **9**, epigallocatechin **10**, or epigallocatechin gallate **11**. In our second experiment, we examined partial substitution of **1 **with ethyl ferulate **12**, feruloyl ethylene glycol **13**, 1-*O*-feruloyl glycerol **14**, 1,3-di-*O*-feruloyl glycerol **15 **or 1,4-di-*O*-feruloyl threitol **16**.

Recent discoveries highlighting the metabolic pliability of plant lignification indicate that lignin can be engineered to dramatically alter its composition. Perturbing single or multiple genes in the monolignol pathway of angiosperms can lead to dramatic shifts in the proportions of normal monolignols (e.g. coniferyl **1 **and sinapyl alcohol **2**, Figure 1) and pathway intermediates polymerized into lignin [1,2]. The malleability of lignification is further illustrated in some angiosperms by the pre-acylation of monolignols with acetate, *p*-hydroxybenzoate, or *p*-coumarate [2,3] and the oxidative coupling of ferulate and diferulate xylan esters into lignin [4-6].

Recent efforts in lignin bioengineering are primarily aimed at manipulating the normal monolignol biosynthetic pathway [7], but in the future apoplastic targeting of phenolics from other metabolic pathways may provide exciting opportunities for designing lignin that is less inhibitory toward polysaccharide hydrolysis and fermentation or easier to remove by biological or chemical pretreatments. Recent model studies with maize cell walls demonstrated that partial substitution of coniferyl alcohol with coniferyl ferulate (a monolignol conjugate) dramatically enhanced the alkaline extractability of lignin and the enzymatic hydrolysis of fiber [8]. Based on these results, bioengineering of plants to copolymerize coniferyl or sinapyl ferulate with monolignols is being pursued as a means for enhancing biomass saccharification or pulping for paper production.

To identify other promising avenues for lignin bioengineering, we are conducting a series of experiments to assess how the inclusion of phenolics derived from various metabolic pathways may alter lignin formation and the utilization of plant cell walls. One path to explore is reducing the hydrophobicity of lignin to permit greater penetration and hydrolysis of fiber by polysaccharidases. Lignin hydrophobicity could be modulated by the incorporation of phenolics with extensive sidechain or aromatic ring hydroxylation (e.g., guaiacyl glycerol **4 **or epigallocatechin gallate **11**) or substitution with hydrophilic groups (e.g., feruloylquinic acid **8 **or 1-*O*-feruloyl glycerol **14**). Another approach would be to incorporate phenolics with *o*-diol functionality (e.g., methyl caffeate **5**, caffeoylquinic acid **6**, epicatechin **9**, epigallocatechin **10**, and epigallocatechin gallate **11**). The presence of *o*-diols provides an intramolecular pathway to trap lignin quinone methide intermediates which form cross-links between lignin and structural polysaccharides [9]; such cross-links appear to limit the enzymatic hydrolysis of cell walls [10,11]. Another route, first illustrated by our work with coniferyl ferulate [8], would be to incorporate readily cleaved bi-phenolic conjugates (e.g., epigallocatechin gallate **11**,1,3-di-*O*-feruloyl glycerol **15**, or 1,4-di-*O*-feruloyl threitol **16**) to facilitate lignin depolymerization during pretreatment of biomass for subsequent saccharification.

In this study, we used a well-characterized biomimetic cell wall model [12] to explore how various monolignol substitutes influence the formation of lignin and the enzymatic degradability of cell walls. Degradability was assessed by monitoring gas production during the in vitro hydrolysis and fermentation of cell walls by rumen microflora and by the analysis of residual nonfermentable polysaccharides (NP). Because gas production is directly linked to structural carbohydrate hydrolysis and fermentation by rumen microflora [13,14] and highly correlated with biomass fermentation to ethanol [15], our findings should have broad application in plant selection and engineering programs aimed at improving the utilization of fibrous feeds by livestock and cellulosic biomass for biofuel production

## Methods

### General

Quinic acid, ferulic acid, and other reagents were obtained from Aldrich. NMR spectra of synthesized compounds were run on a Bruker (Billerica, MA) DRX-360 fitted with a 5 mm 1H/broadband gradient probe with inverse (^1^H-detected) geometry. The solvent used for synthetic compounds was acetone-d_6 _unless otherwise specified; referencing was to the central solvent peak (δ_C _29.80, δ_H _2.04 ppm for acetone).

### Preparation of monolignols and monolignol substitutes

**Figure 2:**
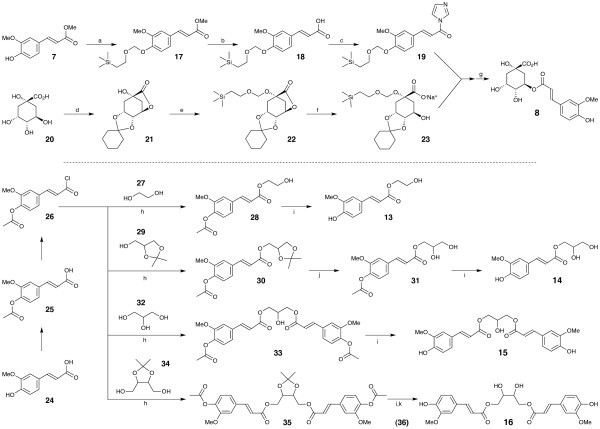
**Synthetic schemes for mono- and diferuloyl compounds 8 and 13-16**. Reagents and conditions are: a. (CH_3_)_3_SiCH_2_CH_2_OCH_2_Cl, [(CH_3_)_2_CH]_2_NH, CH_2_CL_2_; b. 1 M NaOH; c. carbonyldiimidazole, DMF; d. cyclohexanone, DMF; e. 1 M NaOH, H_2_O, dioxane; f. NaH, DMF; g: 1 M HCl; h. CH_2_Cl_2_, pyridine, DMAP; i. pyrrolidine; j. 80% acetic acid; k 1 M HCl.

Coniferyl alcohol **1**, sinapyl alcohol **2**, dihydroconiferyl alcohol **3**, guaiacyl glycerol **4**, methyl ferulate **7**, and ethyl ferulate **12 **were synthesized as described previously [16-19]. Methyl caffeate **5**, caffeoylquinic acid **6**, epicatechin **9**, epigallocatechin **10**, and epigallocatechin gallate **11 **were obtained from commercial sources (Sigma or Indofine). Other mono- and diferuloyl derivatives were synthesized as depicted in Figure 2 and as follows.

#### 5-Feruloylquinic acid 8

The synthesis follows that of chlorogenic acid (caffeoylquinic acid **6**) and other analogs described by Hemmerle et al. [20]. Briefly, methyl ferulate **7 **was protected via reaction with 2-[(trimethylsilyl)ethoxy]methyl chloride to give compound **17 **as an orange oil, which was saponified directly to give **18 **as a solid (58% of theoretical yield based on methyl ferulate); recrystallization from hexane afforded colorless crystals, mp 80-83°C. The amide **19 **was prepared and used directly in coupling with **23**. Protection of (1*R*, 3*R*, 4*R*, 5*R*)-(-)-quinic acid **20 **with cyclohexanone gave solid **21 **in 92% yield. Recrystallization from ether/hexane gave a solid with mp 142.5-143.5°C, lit. [21] mp 142-143°C. Compound **21 **was also protected via reaction with 2-[(trimethylsilyl)ethoxy]methyl chloride to give solid compound **22 **in 95% yield. Recrystallization from hexane gave a colorless solid, mp 103-105°C. The sodium salt **23 **was prepared and used without purification in coupling with compound **19 **to produce the required feruloyl quinic acid **8 **as a foamy solid.

Compound **17**. ^1^H NMR, δ: 0.00 (9H s), 0.94 (2H dd, J = 8.0, 8.3 Hz), 3.72 (3H s), 3.79 (2H dd, J = 8.0, 8.3 Hz), 3.89 (3H s), 5.26 (2H s), 6.44 (1H d, J = 16.0 Hz), 7.13 (1H d, J = 8.3 Hz), 7.18 (1H dd, J = 1.8, 8.3 Hz), 7.33 (1H d, J = 1.8 Hz), 7.60 (1H d, J = 16.0 Hz). ^13^C NMR, δ: -1.3, 18.5, 51.5, 56.2, 66.8, 94.4, 111.9, 116.8, 117.3, 123.0, 129.6, 145.3, 149.9, 151.4, 167.6.

Compound **18**. ^1^H NMR, δ: 0.00 (9H s), 0.94 (2H dd, J = 8.0, 8.3 Hz), 3.79 (2H dd, J = 8.0, 8.3 Hz), 3.89 (3H s), 5.27 (2H s), 6.43 (1H d, J = 16.0 Hz), 7.14 (1H d, J = 8.3 Hz), 7.18 (1H dd, J = 1.7, 8.3 Hz), 7.34 (1H d, J = 1.7 Hz), 7.62 (1H d, J = 16.0 Hz). ^13^C NMR, δ: -1.3, 18.5, 56.2, 66.8, 94.3, 111.9, 117.1, 117.3, 123.0, 129.7, 145.6, 149.8, 151.4, 168.3.

Compound **21**. ^1^H NMR, δ: 1.39 (2H m), 1.53 (2H m), 1.58 (2H m), 1.62 (2H m), 1.71 (2H m), 2.01 (1H dd, J = 14.6, 3.1 Hz), 2.26 (1H dddd, J = 11.7, 6.1, 2.2, 1.4 Hz), 2.34 (1H ddd, J = 11.7, 7.8, 2.2 Hz), 2.51 (1H d, J = 11.7 Hz), 4.29 (1H ddd, J = 6.4, 2.4, 1.4 Hz), 4.53 (1H ddd, J = 7.8, 6.4, 3.1 Hz), 4.65 (1H dd, J = 6.1, 2.4 Hz), 4.95 (1H bs). ^13^C NMR, δ: 24.2, 24.7, 25.7, 34.3, 35.3, 37.6, 39.2, 71.9, 72.0, 72.8, 75.6, 110.6, 178.3.

Compound **22**. ^1^H NMR, δ: 0.02 (9H s), 0.90 (2H m), 3.69 (2H m), 1.40 (2H m), 1.53 (2H m), 1.58 (2H m), 1.64 (2H m), 1.71 (2H m), 2.13 (1H dd, J = 14.6, 3.4 Hz), 2.35 (1H ddd, J = 14.6, 7.7, 2.3 Hz), 2.64 (1H dddd, J = 11.8, 6.4, 2.3, 1.3 Hz), 2.51 (1H d, J = 11.8 Hz), 4.31 (1H ddd, J = 6.4, 2.7, 1.3 Hz), 4.53 (1H ddd, J = 7.7, 6.4, 3.4 Hz), 4.72 (1H dd, J = 6.4, 2.7 Hz), 4.78 (1H d, J = 7.6 Hz), 4.86 (1H d, J = 7.6 Hz). ^13^C NMR, δ: -1.3, 18.5, 24.2, 24.7, 25.7, 32.4, 34.4, 37.7, 37.8, 66.2, 71.8, 72.8, 75.7, 76.6, 91.7, 110.8, 176.1.

Compound **8**. ^1^H NMR (d_4_-methanol), δ: 1.95-2.35 (6H m), 3.78 (1H m), 4.25 (1H m), 5.40 (1H m), 6.38 (1H d, J = 16.0 Hz), 6.86 (1H d, J = 7.8 Hz), 7.12 (1H bd, J = 7.8 Hz), 7.31 (1H bs), 7.59 (1H d, J = 16.0 Hz). ^13^C NMR (d_4_-methanol), δ: 37.8, 39.2, 56.3, 71.2, 71.7, 73.6, 76.2, 111.2, 115.9, 116.0, 123.9, 127.4, 145.7, 148.7, 150.0, 168.3, 175.2.

Compounds **13**-**16 **all derived from coupling reactions between an alcohol and the phenolic acetate of feruloyl chloride **26**, were made from ferulic acid **24**, via the 4-*O*-acetylated ferulic acid **25**, as described previously [22]. Phenolic deacetylation of final products was via pyrrolidine as described previously [23]; the deacetylation to compound **13 **is fully described as an example. The remaining steps were as follows.

#### Feruloyl ethylene glycol 13

2''-Hydroxyethyl-3-(4'-acetoxy-3'-methoxyphenyl)-2-propenoate **28**. 3-(4'-Acetoxy-3'-methoxyphenyl)-2-propenoyl chloride **26 **(1.17 g, 4.6 mmol) and 5 mL of dichloromethane were placed in a 25 mL round-bottom flask equipped with a magnetic stir bar. The mixture was cooled in an ice/water bath. A mixture of 0.02 g of 4-dimethyl-aminopyridine, 0.5 mL of pyridine, and 5 mL of 1,2-dihydroxyethane **27 **were added dropwise to the cooled, stirred acid chloride solution. After the addition, the ice/water bath was removed and the stirred mixture was allowed to warm to ambient temperature. After standing overnight, the mixture was poured into 50 mL of dilute hydrochloric acid (1 M). The mixture was extracted with dichloromethane (3 × 25 mL). The combined organic extracts were washed with saturated sodium bicarbonate solution (1 × 50 mL) and saturated sodium chloride solution (1 × 50 mL) and then dried over anhydrous magnesium sulfate. The solvent was removed using a rotary evaporator to afford 1.12 g of viscous oil (87%). The crude material was deacetylated in the next step without further purification.

Feruloyl ethylene glycol **13 **(2''-Hydroxyethyl-3-(4'-hydroxy-3'-methoxyphenyl)-2-propenoate).

Compound **28 **and 10 mL of pyrrolidine were placed in a 50 mL round-bottom flask and stirred for 10 minutes. The mixture was poured into a separatory funnel containing 75 mL of 1 M hydrochloric acid and 50 mL of ethyl acetate. The pH of the aqueous phase of the mixture was adjusted to below 3 by addition of 1 M hydrochloric acid. The phases were separated and the aqueous phase was extracted with ethyl acetate (3 × 25 mL). The combined organic phases were washed with saturated sodium chloride until the pH was close to neutral (3 × 50 mL). The extracts were dried over anhydrous magnesium sulfate and the solvents removed by rotary evaporator. Removal of residual volatile material on a high-vacuum line afforded 0.838 g of a colorless foam (76% based on the acid chloride **26**).

Compound **28**. ^1^H NMR, δ: 2.24 (3H, s), 3.79 (2H m), 3.89 (3H s), 3.93 (1H t, J = 5.8 Hz), 4.24 (2H dd, J = 4.7, 5.3 Hz), 6.55 (1H d, J = 16.2 Hz), 7.10 (1H d, J = 8.2 Hz), 7.24 (1H dd, J = 1.8, 8.2 Hz), 7.45 (1H d, J = 1.8 Hz), 7.66 (1H d, J = 16.2 Hz). ^13^C NMR, δ: 20.4, 56.4, 60.9, 66.8, 112.4, 119.2, 122.1, 124.1, 142.6, 144.7, 152.7, 167.1, 168.8.

Compound **13**. ^1^H NMR, δ: 3.78 (2H m), 3.91 (3H s), 4.04 (1H bs), 4.22 (3H dd, J = 4.9, 5.1 Hz), 6.37 (1H d, J = 16.0 Hz), 6.86 (1H d, J = 8.2 Hz), 7.11 (1H dd, J = 1.9, 8.2 Hz), 7.31 (1H d, J = 1.9 Hz), 7.60 (1H d, J = 16.0 Hz), 8.27 (1H bs). ^13^C NMR, δ: 56.3, 60.9, 66.5, 111.3, 115.8, 116.1, 123.9, 145.7, 148.8, 150.1, 167.6.

#### 1-O-feruloyl glycerol 14

2,2-Dimethyl-4-(hydroxymethyl)-1,3-dioxolane **29 **from Aldrich (1.36 g, 10.3 mmol), 0.8 g of pyridine, 0.02 g of 4-dimethylaminopyridine, and 20 mL of dichloromethane were placed in a 100 mL round-bottom flask with a magnetic stir bar. The stirred mixture was cooled in an ice/water bath. A solution of 2.55 g (10.0 mmol) of chloride **26 **in 20 mL of dichloromethane was added dropwise to the stirred mixture. After addition, the cooling bath was removed and the mixture was allowed to warm to ambient temperature. The stirring was continued overnight (14 h). The mixture was poured into 20 mL of water contained in a separatory funnel. The phases were separated and the aqueous phase was extracted with dichloromethane (2 × 20 mL). The combined organic phases were washed with saturated ammonium chloride solution (1 × 50 mL), saturated sodium bicarbonate solution (1 × 50 mL), and saturated sodium chloride solution (1 × 50 mL). After drying the organic phase over anhydrous magnesium sulfate, the solvent was removed to afford 3.49 g (99.5%) of **30 **as a white solid. To remove the dioxolane protecting group, compound **30 **was combined with 50 mL of 80% acetic acid in 100 mL round-bottom flask. The mixture was heated on a hot water bath (~90°C) for one hour and then allowed to stand overnight. The mixture was extracted with ethyl acetate (4 × 25 mL). The combined extracts were washed with saturated sodium bicarbonate solution until most of acetic acid was removed (4 × 50 mL) and then washed with saturated sodium chloride solution (1 × 50 mL). The mixture was dried over anhydrous magnesium sulfate. Evaporation of the solvent with a rotary evaporator yielded compound **31 **as a white foam. Phenolic deacetylation of compound **31**, using 20 mL pyrrolidine as described above, yielded 2.61 g of compound **14 **as a white foam (97%).

Compound **30**. ^1^H NMR, δ: 1.31 (3H s), 1.37 (3H s), 2.25 (3H s), 3.79 (1H dd, J = 6.1, 8.4 Hz), 3.89 (3H s), 4.10 (1H dd, J = 6.5, 8.4 Hz), 4.18 (1H dd, J = 6.0, 11.4 Hz), 4.25 (1H dd, J = 4.6, 11.4 Hz), 4.37 (1H m), 6.58 (1H d, J = 16.0 Hz), 7.10 (1H d, J = 8.0 Hz), 7.26 (1H dd, J = 1.7, 8.0 Hz), 7.47 (1H d, J = 1.7 Hz), 7.68 (1H d, J = 16.0 Hz). ^13^C NMR, δ: 20.5, 25.6, 27.1, 56.4, 65.5, 66.9, 74.6, 110.0, 112.4, 118.8, 122.3, 124.1, 134.2, 145.1, 152.7, 166.8, 168.8.

Compound **31**. ^1^H NMR, δ: 2.24 (3H s), 3.60 (2H m), 3.88 (3H s), 3.91 (1H m), 4.18 (1H dd, J = 4.4, 11.3 Hz), 4.27 (1H dd, J = 6.3, 11.3 Hz), 6.56 (1H d, J = 16.3 Hz), 7.10 (1H d, J = 8.2 Hz), 7.25 (1H dd, J = 1.8, 8.2 Hz), 7.46 ( 1H d, J = 8.2 Hz), 7.67 (1H d, J = 16.3 Hz). ^13^C NMR, δ: 20.5, 56.4, 64.0, 65.5, 70.9, 74.5, 112.4, 119.1, 122.1, 124.1, 134.2, 142.7, 144.8, 152.7, 167.1, 168.8.

Compound **14**. ^1^H NMR (d_6_-acetone), δ 3.61 (2H m), 3.89 (1H m), 3.90 (3H s), 4.21 (2H m), 6.38 (1H d, J = 16.0 Hz), 6.86 (1H d, J = 8.2 Hz), 7.13 (1H bd, J = 8.2 Hz), 7.32 (1H bs), 7.61, (1H d, J = 16.0 Hz), 8.12 (1H bs). ^13^C NMR (d_6_-acetone), δ 56.3, 64.1, 66.3, 70.9, 111.3, 115.7, 116.1, 124.1, 127.4, 145.8, 148.7, 150.1, 167.6.

#### 1,3-di-O-feruloyl glycerol 15

Chloride **26 **(2.55 g, 10.0 mmol) and 5 mL of dichloromethane were placed in a 25 mL round-bottom flask equipped with a magnetic stir bar. The mixture was cooled in an ice/water bath. A mixture of 0.02 g of 4-dimethyl-aminopyridine, 0.9 mL of pyridine, and 0.496 g of glycerol **32 **was added dropwise to the cooled, stirred acid chloride solution. After the addition, the ice/water bath was removed and the stirred mixture was allowed to warm to ambient temperature. After standing overnight, the mixture was poured into 50 mL of dilute hydrochloric acid (1M). The mixture was extracted with dichloromethane (3 × 25 mL). The combined organic extracts were washed with saturated sodium bicarbonate solution (1 × 50 mL) and saturated sodium chloride solution (1 × 50 mL) and then dried over anhydrous magnesium sulfate. The solvent was removed using a rotary evaporator to afford 2.16 g of compound **33 **as a viscous oil (82%). The crude material and 20 mL of pyrrolidine were placed in a 50 mL round-bottom flask and stirred for 10 minutes to effect deacetylation. Workup as described above yielded 1.65 g of product **15 **as a viscous oil. The material was purified by flash chromatography on silica gel (30% ethyl acetate in hexanes) to afford 0.724 g of amorphous solid (32%).

Compound **33**. ^1^H NMR, δ: 2.24 (6H s), 3.87 (6H s), 4.21 (1H m), 4.31 (4H m), 6.58 (2H d, J = 16. 0 Hz), 7.09 (2H d, J = 8.2 Hz), 7.24 (2H dd, J = 1.6, 8.2 Hz), 7.43 (2H d, J = 1.6 Hz), 7.69 (2H d, J = 16.0 Hz). ^13^C NMR, δ: 20.4, 56.4, 66.2, 68.2, 112.4, 118.9, 122.2, 124.1, 134.2, 142.7, 145.0, 152.7, 167.0, 168.8.

Compound **15**. ^1^H NMR, δ: 3.89 (6H s), 4.18 (1H m), 4.27 (4H m), 6.41 (2H d, J = 15.9 Hz), 6.86 (2H d, J = 8.2 Hz), 7.12 (2H dd, J = 1.8, 8.2 Hz), 7.31 (2H d, J = 1.8 Hz), 7.63 (2H d, J = 15.9 Hz). ^13^C NMR, δ: 56.3, 66.0, 68.3, 111.2, 115.4, 116.1, 124.0, 127.3, 146.0, 148.8, 167.4.

#### 1,4-di-O-feruloyl threitol 16

2,3-Di-*O*-isopropylidene-L-threitol **34 **was prepared by the method described in Organic Syntheses, Coll. Vol. VIII, p. 155, 1993. Compound **34 **(1.01 g, 6.2 mmol), 0.01 g of 4-dimethylpyridine, 1.2 mL of pyridine, and 10 mL of 1,2-dichloroethane were placed in a 100 mL round-bottom flask with a magnetic stir bar. The mixture was cooled in an ice/water bath. A solution of 3.46 g (13.6 mmol) of chloride **26 **in 25 mL of dichloromethane was added dropwise to the stirred mixture. After addition, the cooling bath was removed and the mixture was allowed to warm to ambient temperature and the mixture was allowed to stand 14 h. The mixture was poured into 20 mL of water contained in a separatory funnel. The phases were separated and the aqueous phase was extracted with dichloromethane (2 × 25 mL). The combined organic phases were washed with saturated ammonium chloride solution (2 × 50 mL), saturated sodium bicarbonate solution (2 × 50 mL), and saturated sodium chloride solution (1 × 50 mL). After drying the organic phase over anhydrous magnesium sulfate, the solvent was removed to afford 3.48 g (93%) of compound **35 **as a white foam. Trituration of the foam with methanol yielded 2.71 g of white crystals. Deacetylation of compound **35 **(2.01 g, 3.4 mmol) with 20 mL of pyrrolidine, as above, yielded 1.77 g of the deacetylated **35**, compound **36**, as a white foam (100%). The material from above was dissolved in 25 mL of 1,4-dioxane in 125 mL Erlenmeyer flask. Fifty mL of 2N hydrochloric acid was added to remove the dioxolane protecting group, and the mixture was stirred using a magnetic stirrer. Cloudiness in the mixture was removed by dropwise addition of 1,4-dioxane. After stirring overnight at ambient temperature, the mixture was extracted with ethyl acetate (4 × 25 mL). The combined extracts were washed with saturated sodium bicarbonate solution until most of acetic acid was removed (4 × 50 mL) and then washed with saturated sodium chloride solution (1 × 50 mL). The mixture was dried over anhydrous magnesium sulfate. Evaporation of the solvent with a rotary evaporator yielded 1.37 g of compound **16 **as a white foam (85%).

Compound **35**. ^1^H NMR, δ: 1.41 (6H s), 2.25 (6H s), 4.24 (2H m), 4.35 (2H dd, J = 5.2, 11.7 Hz), 4.48 (2H dd, J = 3.2, 11.7 Hz), 6.60 (2H d, J = 16.0 Hz), 7.09 (2H d, J = 8.2 Hz), 7.24 (2H dd, J = 1.7, 8.2 Hz), 7.43 (2H d, J = 1.7 Hz), 7.69 (2H d, J = 16.0 Hz). ^13^C NMR, δ: 20.4, 27.32, 56.3, 64.7, 76.9, 11-.6, 112.5, 118.6, 122.2, 124.1, 134.0, 142.7, 145.3, 152.6, 166.7, 168.8.

Compound **36**. ^1^H NMR, δ: 1.40 (6H s), 3.90 (6H s), 4.22 (2H m), 4.32 (2H m), 4.44 (2H m), 6.43 (2H d, J = 15.9 Hz), 6.87 (2H d, J = 8.2 Hz), 7.14 (2H dd, J = 1.8, 8.2 Hz), 7.33 (2H d, J = 1.8 Hz), 7.64 (2H d, J = 15.9 Hz), 8.43 (2H bs). ^13^C NMR, δ: 27.3, 56.3, 64.5, 77.0, 110.5, 111.4, 115.2, 124.0, 127.3, 146.3, 148.7, 150.2, 167.2.

Compound **16**. ^1^H NMR, δ: 3.89 (6H s), 4.01 (2H t, J = 5.1 Hz), 4.32 (4H d, J = 5.7), 6.39 (2H d, J = 16.0 Hz), 6.86 (2H d, J = 8.3 Hz), 7.11 (2H dd, J = 1.6, 8.3 Hz), 7.30 (2H d, J = 8.3 Hz), 7.61 (2H d, J = 16 Hz), 8.15 (2H bs). ^13^C NMR, δ: 56.3, 66.0, 70.2, 111.3, 115.6, 116.1, 123.8, 127.4, 145.9, 148.7, 150.1, 167.5.

### Cell Wall Lignification

Nonlignified primary cell walls (~1.8 g dry weight) isolated from maize cell suspensions [24] were stirred in 250 mL of Homopipes buffer (75 mM, pH 5.5) containing 2000 units of glucose oxidase (Sigma, EC 1.1.3.4). Glucose (0.25 mmol in 2.5 mL of water) was added dropwise to cell wall suspensions over 30 min and then stirred for 30 min to generate H_2_O_2 _(~2 eq/mol of cell wall ferulate) for dimerizing ferulates by wall-bound peroxidases. Cell walls were then artificially lignified by adding a two-component mixture of coniferyl alcohol **1 **(0.6 mmol) and sinapyl alcohol **2 **(0.6 mmol) or three-component mixtures of coniferyl alcohol (0.4 mmol) and sinapyl alcohol (0.4 mmol) with one of the following monolignol substitutes: dihydroconiferyl alcohol **3 **(0.4 mmol), guaiacylglycerol **4 **(0.4 mmol), methyl caffeate **5 **(0.4 mmol), caffeoylquinic acid **6 **(0.4 mmol), methyl ferulate **7 **(0.4 mmol), feruloylquinic acid **8 **(0.4 mmol), epicatechin **9 **(0.4 mmol), epigallocatechin **10 **(0.4 mmol), or epigallocatechin gallate **11 **(0.2 mmol). The monolignol mixtures and glucose (1.5 mmol, to generate H_2_O_2_), prepared in 2.5 mL dioxane and 95 mL of water, were added dropwise to the cell wall suspensions over a ~20 h period.

In a separate study, nonlignified primary cell walls (~2 g dry weight) isolated from maize cell suspensions [24] were stirred in 300 mL of Homopipes buffer (25 mM, pH 5.5) and H_2_O_2 _(0.35 mmol in 3.5 mL of water, ~3 eq/mol of cell wall ferulate) was added dropwise over 30 min to dimerize ferulates by wall-bound peroxidases. After stirring for an additional 30 min, cell walls were artificially lignified with coniferyl alcohol **1 **(1.2 mmol) or lignified with a two-component mixture of coniferyl alcohol (0.8 mmol) with one of the following monolignol substitutes: ethyl ferulate **12 **(0.4 mmol), feruloyl ethylene glycol **13 **(0.4 mmol), 1-*O*-feruloyl glycerol **14 **(0.4 mmol), 1,3-di-*O*-feruloyl glycerol **15 **(0.2 mmol) or 1,4-di-*O*-feruloyl threitol **16 **(0.2 mmol). Separate solutions of monolignol mixtures (prepared in 16 mL dioxane and 44 mL of water) and H_2_O_2 _(1.4 mmol, prepared in 60 mL of water) were added dropwise to the cell wall suspensions over a ~20 h period. In this experiment, a higher proportion of dioxane was used to prepare all monolignol solutions because feruloyl ethylene glycol and 1,3-di-*O*-feruloyl glycerol were observed to have poor solubility in water.

In both studies, treatments were replicated twice in independent experiments and nonlignified controls were stirred in a solvent mixture similar to the final makeup of the lignification reaction media. Cell wall peroxidase activity at the end of monolignol addition was visually assessed with guaiacol-H_2_O_2 _staining [25]. Following additions, cell walls were stirred for an additional 24 h, pelleted (5,000 × *g*, 15 min) and twice resuspended in 600 mL of water for 60 min, and pelleted (5,000 × *g*, 15 min) to remove low molecular weight dehydrogenation products. The cell wall pellets were then resuspended in 650 mL of 9:1 (v/v) acetone:water for 30 min, collected on glass-fiber filters (1.2 μm retention) and washed with 300 mL of acetone:water to remove non-bound lignins. After repeated washing with acetone, cell walls were set overnight in a hood to evaporate off acetone and then dried at 55°C and weighed.

### Cell wall analyses

Acid-insoluble Klason lignin in cell wall samples (75 mg) was determined in duplicate by a two-stage hydrolysis in 12 M H_2_SO_4 _at 25°C for 2 h followed by 1.6 M H_2_SO_4 _at 100°C for 3 h [26]. Whole cell walls (~50 mg) from selected lignification treatments were sonicated in DMSO-d_6 _with pyridine-d_5 _and subjected to gel-state 2-D NMR using a Bruker-Biospin (Billerica, MA) 500 MHz Avance spectrometer equipped with an inverse gradient 5-mm TCI cryoprobe, as previously described [27].

Gas production during microbial hydrolysis and fermentation of cell walls (100 mg) at 39°C in 60 mL sealed bottles was monitored with pressure transducers for 45 h following addition of 5.7 mL of phosphate-bicarbonate buffer, 0.3 mL of reducing agent, and 4 mL of diluted rumen inoculum [28]. Filtered inoculum was prepared with a 1:2 ratio (v/v) of rumen fluid and blended buffer-extracted rumen solids collected from two Holstein cows fed a total mixed ration of corn silage, corn grain, alfalfa hay, soybean meal, and supplemental vitamins and minerals [28]. Blank-corrected gas production data from two to four independent fermentation runs were fitted with a dual-pool logistic model to estimate the kinetics of microbial hydrolysis and fermentation of cell walls [28]. Freeze-dried residues remaining after cell-wall degradation by rumen microflora were dissolved in 12 M H_2_SO_4 _at 25°C for 2 h and analyzed for NP by the phenol-sulfuric acid assay [29] with corrections for inoculum contamination and sugar recovery. The recovery of sugars from NP was estimated by running unfermented nonlignified cell walls through the 12 M H_2_SO_4 _dissolution/phenol-sulfuric acid assay procedure.

### Statistical methods

Data for monolignol treatments were subjected to an analysis of variance by PROC GLM according to a randomized complete block design with two replications [30,31]. If *F*-tests were significant (*P *≤ 0.05), then differences among monolignol treatment means were tested by the LSD procedure (*P *= 0.05). Unless otherwise noted, all reported treatment differences were significant at *P *= 0.05.

## Results and discussion

### Cell wall lignification

Previous work demonstrated that artificial lignins formed in primary cell walls isolated from maize cell suspensions are structurally similar to those naturally formed in grasses [24]. In the current study, isolated maize cell walls containing bound peroxidases were artificially lignified with normal monolignols (coniferyl and sinapyl alcohols **1**, **2**) added alone or in combination with various phenolic monolignol substitutes **3**-**16**. Each monolignol substitute comprised about one-third by weight of the precursor mixture, a shift in lignin composition comparable to that often observed in mutant or transgenic plants with altered lignin biosynthesis. After dissolution in a minimal volume of dioxane, normal monolignols and most monolignol substitutes readily formed aqueous solutions, but ethyl ferulate **12 **and polyol esters, especially feruloyl ethylene glycol **13 **and 1,3-di-*O*-feruloyl glycerol **15**, precipitated unless a high proportion of dioxane (~25%) was used.

At the conclusion of lignification, guaiacol staining indicated weak residual peroxidase activity following addition of normal monolignols with monoferuloyl esters, very weak to no activity with diferuloyl esters, but good activity for all other treatments (data not shown). While peroxidase activity is gradually lost during lignification [32], partial substitution of monolignols with mono- or especially diferuloyl esters greatly accelerated this process. Among possible inactivation pathways [33-35], active site attack by ferulate phenoxy radicals or enzyme precipitation due to sorption or cross-linking to polymeric products are most plausible given the extremely high substrate preference of maize peroxidase for ferulate esters [36], the bi-phenolic cross-linking capability of diferuloyl polyol esters, and their aforementioned poor aqueous solubility.

**Figure 3:**
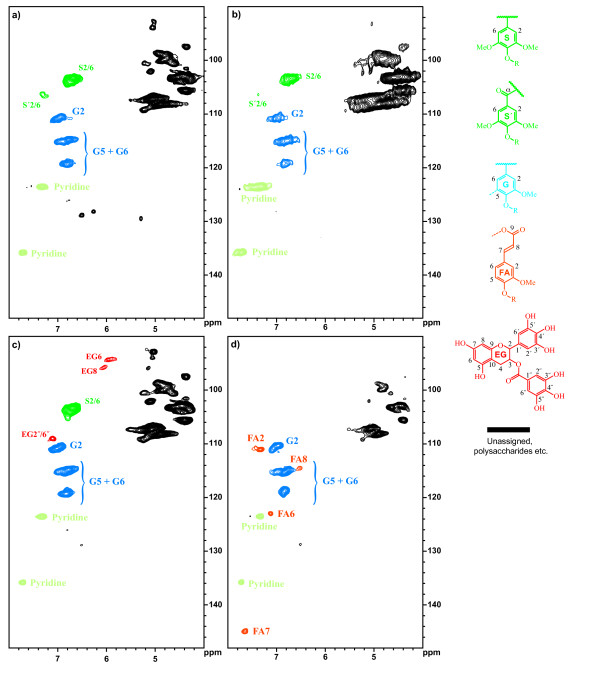
**Aromatic regions from **^**13**^**C-**^**1**^**H correlation gel-state 2D NMR spectra (HSQC) of whole cell walls in DMSO-d**_**6 **_**and pyridine-d**_**5**_. Maize cell walls were artificially lignified with a) coniferyl alcohol **1 **and sinapyl alcohol **2 **in a 1:1 molar ratio, b) coniferyl alcohol, sinapyl alcohol, and feruloylquinic acid **8 **in a 1:1:1 molar ratio, c) coniferyl alcohol, sinapyl alcohol, and epigallocatechin gallate **11 **in a 1:1:0.5 molar ratio, and d) coniferyl alcohol and 1,4-di-*O-*feruloyl threitol **16 **in a 4:1 molar ratio. Correlations for syringyl (S), guaiacyl (G), ferulate (FA), and epigallocatechin (EG) units in lignin were assigned using data from previous publications [53-55] and an NMR database [56].

Based on Klason lignin analysis, inclusion of feruloylquinic acid **8**, methyl caffeate **5**, or especially caffeoylquinic acid **6 **considerably depressed lignin formation while methyl ferulate **7 **readily copolymerized with monolignols to form wall bound lignin (Table 1). Methyl and quinic acid esters of ferulic and caffeic acids are plant extractives, of which caffeoylquinic acid (also known as chlorogenic acid) and feruloylquinic acid are especially abundant in coffee and some other beverages and foods [37]. To tentatively investigate the cause of depressed lignification, cell walls from selected treatments were subjected to gel-state 2D NMR. Spectra of cell walls lignified with normal monolignols (Figure 3a) or monolignols plus feruloylquinic acid (Figure 3b) both contained only correlations of guaiacyl units (derived from coniferyl alcohol) and syringyl units (derived from sinapyl alcohol); the absence of ferulate correlations in the latter spectrum suggests that feruloylquinic acid was not incorporated into wall bound lignin. It must, however, be pointed out that correlations of native cell wall ferulates comprising ~10% of the lignin polymer are also missing from spectra of control cell walls (Figure 3a). Thus extensive polymerization of ferulates into a wide array of coupling products [4-6] may sufficiently disperse their correlations or coalesce them with adjacent guaiacyl correlations to render them undetectable in our 2D NMR experiments. In any case, it seems that depressed lignin formation with these monolignol substitutes was associated with the presence of quinic or caffeic acid moieties. Highly hydrophilic quinic acid moieties might hinder the association and copolymerization of hydroxycinnamate radicals with hydrophobic lignin polymers, but this or alternative mechanisms require further study. Peroxidase-generated radicals of caffeate can undergo homo-dimerization or cross-coupling reactions with ferulate and sinapate [38], but in the current study any coupling products involving caffeate apparently did not become incorporated into polymeric wall-bound lignin. Alternatively, poor incorporation of caffeate into lignin may be due to its tendency to form quinones, which avoid radical coupling reactions characteristic of monolignols [39]; to our knowledge oxidative coupling reactions between caffeate and monolignols have not been reported and call for additional study.

**Table 1:** Klason lignin, in-vitro ruminal fermentation kinetics^a^, nonfermentable polysaccharides (NP), gas reduction per unit lignin (GRL)^b^, and nonfermentable polysaccharide accumulation per unit lignin (NPAL)^c ^for nonlignified and artificially lignified cell walls of maize.

Monolignols	Lignin mg/g	*L*_1 _(h)	*k*_1 _(h^-1^)	*A *(mL/g)	*L*_2 _(h)	*k*_2 _(h^-1^)	*B *(mL/g)	*AB *(mL/g)	NP (mg/g)	GRL	NPAL
Nonlignified	--	2.0	0.217	290	2.4	0.044	53	343	22	--	--
CA:SA	153	3.4	0.097	243	19.9	0.067	17	259	114	0.545	0.604
CA:SA:DHCA	154	3.0	0.094	241	17.6	0.068	23	265	113	0.508	0.594
CA:SA:GG	152	2.6	0.099	243	13.6	0.053	28	271	94	0.473	0.474
CA:SA:MC	111	2.1	0.124	265	9.1	0.049	33	298	59	0.406	0.343
CA:SA:CQA	92	2.1	0.160	272	5.3	0.050	42	315	28	0.308	0.071
CA:SA:MF	159	3.2	0.080	211	15.6	0.067	23	235	166	0.680	0.911
CA:SA:FQA	117	2.8	0.130	265	10.7	0.048	30	295	52	0.410	0.258
CA:SA:EC	170	2.9	0.060	238	31.6	0.118	6	244	155	0.578	0.779
CA:SA:EGC	160	3.1	0.094	259	23.3	0.089	11	270	87	0.455	0.413
CA:SA:EGCG	167	3.1	0.098	246	16.6	0.057	23	269	88	0.443	0.399
LSD^d^	12	0.7	0.015	16	9.9	0.036	17	14	21	0.115	0.120

^a^Kinetic parameters: lag time (*L*_1_), rate constant (*k*_1_), and volume (*A*) of gas produced from a rapidly digested pool; lag time (*L*_2_), rate constant (*k*_2_), and volume (*B*) of gas produced from a slowly digested pool; and total gas volume (*AB*)

^b^GRL calculated as (*AB*_nonlignified _- *AB*_lignified_)/Klason lignin

^c^NPAL calculated as (NP_lignified _- NP_nonlignified_)/Klason lignin

^d^LSD, least significant difference (*P *= 0.05).

Cell walls were lignified with a binary mixture of coniferyl alcohol (CA) and sinapyl alcohol (SA) or trinary mixtures of CA and SA with dihydroconiferyl alcohol (DHCA), guaiacylglycerol (GG), methyl caffeate (MC), caffeoylquinic acid, (CQA), methyl ferulate (MF), feruloylquinic acid (FQA), epicatechin (EC), epigallocatechin (EGC), or epigallocatechin gallate (EGCG).

In contrast to caffeate, Klason lignin analysis suggested other benzene diol or triols (epicatechin **9**, epigallocatechin **10**, and epigallocatechin gallate **11**) readily formed copolymer lignins with monolignols (Table 1). Indeed, the incorporation of catechins into lignin is illustrated by diagnostic 2D gel NMR contours for epigallocatechin gallate (Figure 3c.) The catechins, which are precursors of vacuolar-deposited tannins in plants, readily undergo radical homo-coupling reactions [40-42]. Their coupling reactions with monolignols are, however, unknown and require further study. Dihydroconiferyl alcohol **3 **and guaiacylglycerol **4**, differing in sidechain hydroxylation, were also extensively copolymerized into lignin. These phenylpropanoids are known to cross-couple with monolignols via their phenolic ring to form endgroups in softwood lignins [1].

In contrast to feruloylquinic acid, Klason lignin analysis indicated excellent incorporation of other feruloyl esters involving ethyl **12**, ethylene glycol **13**, or glycerol **14 **groups (Table 2), despite their tendency to accelerate peroxidase inactivation. As noted previously with hydroxycinnamate-monolignols esters [8,25], diferuloyl polyols **15 **and **16 **depleted peroxidase activity and depressed lignification, but they became substantial components of lignin based on diagnostic ferulate correlations in NMR spectra (e.g., Figure 3d). Oxidative coupling reactions of feruloyl polyol esters have not been reported, but they would likely form a wide array of ferulate, diferulate, and ferulate-monolignol structures similar to those previously observed with ethyl ferulate, ferulate-monolignol conjugates, and various ferulate-polysaccharide esters [4,5,8,43-45].

**Table 2:** Klason lignin, in-vitro ruminal fermentation kinetics^a^, nonfermentable polysaccharides (NP), gas reduction per unit lignin (GRL)^b^, and nonfermentable polysaccharide accumulation per unit lignin (NPAL)^c ^for nonlignified and artificially lignified cell walls of maize.

Monolignols	Lignin mg/g	*L*_1 _(h)	*k*_1 _(h^-1^)	*A *(mL/g)	*L*_2 _(h)	*k*_2 _(h^-1^)	*B *(mL/g)	*AB *(mL/g)	NP (mg/g)	GRL	NPAL
Nonlignified	--	1.83	0.244	298	0.4	0.033	67	365	21	--	--
CA	151	1.95	0.099	231	11.1	0.043	46	277	130	0.589	0.708
CA:EF	154	1.94	0.082	225	14.9	0.037	35	260	162	0.697	0.907
CA:FEG	149	2.44	0.092	231	12.5	0.041	40	271	142	0.647	0.808
CA:FG	149	2.31	0.114	221	9.5	0.040	48	269	122	0.652	0.676
CA:DFG	124	2.33	0.135	251	9.4	0.040	47	299	81	0.544	0.487
CA:DFT	135	2.36	0.117	236	10.3	0.040	43	279	90	0.650	0.508
LSD^d^	20	0.51	0.017	23	1.9	0.008	12	30	38	NS ^e^	0.163

^a^Kinetic parameters: lag time (*L*_1_), rate constant (*k*_1_), and volume (*A*) of gas produced from a rapidly digested pool; lag time (*L*_2_), rate constant (*k*_2_), and volume (*B*) of gas produced from a slowly digested pool; and total gas volume (*AB*)

^b^GRL calculated as (*AB*_nonlignified _- *AB*_lignified_)/Klason lignin

^c^NPAL calculated as (NP_lignified _- NP_nonlignified_)/Klason lignin

^d^LSD, least significant difference (*P *= 0.05)

^e^NS, not significant (*P = 0.43*).

Cell walls were lignified with coniferyl alcohol (CA) or binary mixtures of CA with ethyl ferulate (EF), feruloyl ethylene glycol (FEG), 1-*O*-feruloyl glycerol (FG), 1,3-*O*-diferuloyl glycerol (DFG), or 1,4-*O*-diferuloyl threitol (DFT).

### Cell wall hydrolysis and fermentation by rumen microflora

Because maize cell walls used in our lignification experiments were fully disaggregated and extremely thin (~1 μm), they exposed a large surface area that could be quickly colonized by rumen microflora [24]. Furthermore, rumen microflora produce a diverse array of hydrolases capable of rapidly degrading essentially all organic components except lignin [46]. Together, these factors allowed us assess how lignin alterations affect cell wall degradation at the molecular level, free from other confounding constraints that also hinder cell wall degradation such as the anatomical structure of plant tissues or the use of enzyme preparations lacking key hydrolase activities [12]. In addition to measuring residual NP at the endpoint of digestion, we continuously monitored gas production by rumen bacteria to estimate lag time before fermentation commenced as well as the rate and extent of cell wall fermentation. Although the production of fermentation gasses by rumen bacteria is substrate dependent [14], our use of a common source of cell walls for all lignification treatments insured that shifts in gas production mirrored the enzymatic degradation of cell walls.

As in a previous study [47], cell walls exhibited a biphasic production of fermentation gasses that was best described by a two-pool logistic model with two discrete lag times. For nonlignified cell walls, gas production for both pools commenced after a lag of <2.5 h, but the primary pool had roughly a 5-fold greater rate and extent of gas production than the secondary pool (Table 1 and 2). Nonlignified cell walls were extensively degraded, leaving only 21 mg/g of NP. Based on the polysaccharide composition and fermentation characteristics of primary maize walls, we previously speculated the larger pool represented rapidly degraded noncellulosic polysaccharides, while the smaller pool represented slowly degraded cellulose [47]. The relative rate and extent of gas production for these pools were, however, strikingly similar to two fiber digestion pools observed in graminaceous crops [48], which undoubtedly possess a markedly different structural polysaccharide makeup than our primary maize cell walls. Clearly, further work is warranted to reveal the exact structural nature of the rapidly and slowly degraded pools in grass cell walls.

In both experiments, total gas *AB *declined 24% and NP increased by ~100 mg/g when cell walls were artificially lignified with normal monolignols (coniferyl and sinapyl alcohols **1**, **2**) to a Klason lignin content of 150 mg/g (Table 1 and 2). Lignification with normal monolignols had little effect on lag *L*_*1*_, but rate *k*_*1 *_and gas production *A *for the large rapidly degraded pool decreased by 57 and 24%, respectively. Conversely for the smaller pool, lignification with normal monolignols dramatically increased lag *L*_*2 *_by 10.7 to 17.5 h and decreased gas production *B *by 30 to 70%, with comparatively minor effects on rate *k*_*2*_. Although effects on the rapidly and slowly degraded pools differed, the overall impact of lignification was to obstruct cell wall hydrolysis and fermentation by rumen bacteria. Similar findings were previously reported in studies utilizing coarsely ground grass stems and isolated tissues from grasses [49,50]. Thus, lignification is an impediment to the enzymatic hydrolysis and fermentation of cell walls at both the molecular and tissue level in plants.

**Figure 4:**
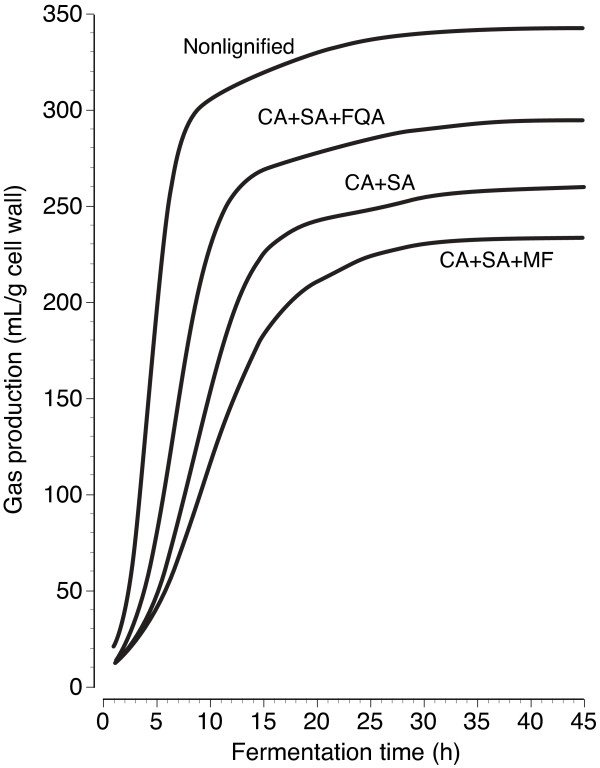
**Blank-corrected in vitro gas production curves from nonlignified and artificially lignified cell walls incubated with rumen microflora**. Artifically lignified cell walls were prepared with a 1:1 molar ratio of coniferyl alcohol plus sinapyl alcohol (CA+SA), or a 1:1:1 molar ratio of CA and SA plus feruloylquinic acid (CA + SA + FQA) or methyl ferulate (CA + SA + MF).

In the first experiment, substituting one-third of normal monolignols with methyl caffeate **5**, caffeoylquinic acid **6**, or feruloylquinic acid **8 **strikingly improved total gas *AB *and reduced NP for lignified cell walls, primarily through increasing rate *k*_*1 *_and gas production *A *from the large rapidly digested pool (Table 1). These monolignol substitutes also reduced lag times for both pools, particularly lag *L*_*2 *_of the small slowly digested pool. The impact of these shifts on gas production from cell walls lignified with feruloylquinic acid is shown in Figure 4. These monolignol substitutes also lessened gas reduction per unit lignin (GRL) and nonfermentable polysaccharide accumulation per unit of lignin (NPAL), indicating that factors in addition to reduced lignin content contributed to enhanced cell wall hydrolysis and fermentation (Table 1). Caffeate, like other benzene-1,2-diols, might internally trap lignin quinone-methide intermediates [51,52] to limit the cross-linking of lignin to polysaccharides and enhance the enzymatic hydrolysis of cell walls [10,11]. Incorporation of hydrophilic caffeoyl or quinic acid moieties into lignin might also enhance penetration or limit irreversible binding of hydrolytic enzymes to the lignocellulosic matrix, but identifying actual causative factors requires further study.

While not conclusively shown by the kinetics of gas production, reductions in NP and NPAL indicated a modest improvement in cell wall hydrolysis occurred when one-third of normal monolignols were substituted with guaiacylglycerol **4**, epigallocatechin **10**, or epigallocatechin gallate **11 **(Table 1). In contrast, dihydroconiferyl alcohol **3 **had no effect, while incorporation of epicatechin **9 **and especially methyl ferulate **7 **adversely affected cell wall hydrolysis and fermentation (Figure 4) by decreasing rate *k*_*1 *_and gas production *A *and *AB*, and by increasing NP, GRL, and NPAL. Among the catechins, those with 1,2,3-triol functionality enhanced cell wall hydrolysis and fermentation, primarily by increasing rate *k*_*1 *_and gas production *AB *and by decreasing lag *L*_*2*_, NP, GRL, and NPAL--again implicating reduced hydrophobicity or cross-linking as playing a role--but underlying mechanisms await revelation. In contrast, increased sidechain hydroxylation of incorporated phenylpropanoids (i.e., dihydroconiferyl alcohol vs. guaiacylglycerol) slightly increased cell wall hydrolysis as indicated by modest reductions (*P <*0.1) in NP or NPAL.

In the second experiment, substituting one-third of a normal monolignol (coniferyl alcohol) with various monoferuloyl esters did not alter cell wall hydrolysis and fermentation (Table 2). But within the monoferuloyl ester treatments, increases in ester group hydroxylation slightly increased rate *k*_*1 *_and a reduced lag *L*_*2*_, NP and NPAL. Incorporating 1,4-di-*O*-diferuloyl threitol **16 **and particularly1,3-di-*O*-feruloyl glycerol **15 **into lignin, however, modestly increased rate *k*_*1 *_and reduced both NP and NPAL compared to cell walls lignified solely with coniferyl alcohol. While having relatively small effects on the inherent degradability of non-pretreated cell walls, incorporation of diferuloyl polyol esters (and other readily cleaved bi-phenolic conjugates like epigallocatechin gallate) could substantially boost pretreatment efficacy for removing lignin prior to biomass saccharification. Other biomimetic model studies by our group recently demonstrated the potential for bi-phenolic conjugates to improve cell wall delignification and saccharification [8].

## Conclusion

To identify new targets for lignin bioengineering, we artificially lignified maize cell walls with normal monolignols and a variety of phenolic monolignol substitutes normally associated with other plant metabolic pathways. Lignification with normal monolignols severely impeded structural polysaccharide hydrolysis and fermentation by rumen microflora at the molecular level in cell walls. Inclusion of feruloylquinic acid, methyl caffeate, or caffeoylquinic acid with normal monolignols considerably depressed lignin formation and substantially improved cell wall hydrolysis and fermentation. In contrast, epicatechin, epigallocatechin, and epigallocatechin gallate readily formed copolymer lignins with monolignols as did the phenylpropanoids dihydroconiferyl alcohol and guaiacylglycerol. Cell wall fermentability was moderately enhanced by catechins with 1,2,3-triol functionality, while inclusion of phenylpropanoids with extensive sidechain hydroxylation had little impact. Mono- or diferuloyl esters with various aliphatic or polyol groups readily formed copolymers with monolignols, but their tendency to accelerate peroxidase inactivation slightly diminished lignin formation in some cases. Relative to cell walls lignified with normal monolignols, copolymerization of mono- or diferuloyl esters into lignin had negative to weakly positive effects on cell wall hydrolysis and fermentability, which were in part dependent on the quantity of lignin formed and the degree of ester group hydroxylation. Overall, monolignol substitutes improved the inherent degradability of cell walls by depressing lignin formation or possibly by reducing lignin hydrophobicity or cross-linking to structural polysaccharides. Some monolignol substitutes, chiefly readily cleaved bi-phenolic conjugates like epigallocatechin gallate or diferuloyl polyol esters are expected to greatly boost enzymatic saccharification of cell walls following chemical pretreatment. In a forthcoming paper, we will examine the impact of the aforementioned monolignol substitutes on the enzymatic saccharification of intact and chemically pretreated cell walls. In subsequent model studies, we will examine these and other monolignol substitutes in greater detail to identify promising bioengineering targets for improving plant fiber utilization.

## Competing interests

The authors declare that they have no competing interests.

## Authors' contributions

Using input from coauthors, JG designed and carried out lignification experiments, characterized the lignin content and degradability of cell walls, and led the writing of the manuscript. PS developed methods for preparing feruloylquinic acid and a series of mono- and diferuloyl polyol esters and with JR, prepared Figure 2 and described their syntheses. Using previously developed methods, HK and FL contributed monolignols and other monolignol substitutes examined in this study. HK and JR carried out gel 2D-NMR analyses of cell walls. JR helped to coordinate the project and edited the final manuscript. All authors read and accepted the final manuscript.

## References

[B1] Boerjan W Ralph J Baucher M Lignin biosynthesis Annual Rev Plant Biol 2003 54 519 546 10.1146/annurev.arplant.54.031902.134938 14503002

[B2] Ralph J Lundquist K Brunow G Lu F Kim H Schatz PF Marita JM Hatfield RD Ralph SA Christensen JH Boerjan W Lignins: Natural polymers from oxidative coupling of 4-hydroxyphenylpropanoids Phytochem Rev 2004 3 29 60 10.1023/B:PHYT.0000047809.65444.a4

[B3] Lu F Ralph J Novel tetrahydrofuran structures derived from β-β-coupling reactions involving sinapyl acetate in kenaf lignins Org Biomol Chem 2008 6 3681 3694 10.1039/b809464k 18843398

[B4] Grabber JH Ralph J Hatfield RD Cross-linking of maize walls by ferulate dimerization and incorporation into lignin J Agric Food Chem 2000 48 6106 6113 10.1021/jf0006978 11312783

[B5] Grabber JH Ralph J Hatfield RD Model studies of ferulate-coniferyl alcohol cross-product formation in primary maize walls: Implications for lignification in grasses J Agric Food Chem 2002 50 6008 6016 10.1021/jf0205312 12358473

[B6] Ralph J Bunzel M Marita JM Hatfield RD Lu F Hoon K Schatz PF Grabber JH Steinhart H Peroxidase-dependent cross-linking reactions of *p*-hydroxycinnamates in plant cell walls Phytochem Rev 2004 3 79 96 10.1023/B:PHYT.0000047811.13837.fb

[B7] Vanholme R Morreel K Ralph J Boerjan W Lignin engineering Curr Opin Plant Biol 2008 11 1 8 10.1016/j.pbi.2008.03.005 18434238

[B8] Grabber JH Hatfield RD Lu F Ralph J Coniferyl ferulate incorporation into lignin enhances the alkaline delignification and enzymatic degradation of cell walls Biomacromolecules 2008 9 2510 2516 10.1021/bm800528f 18712922

[B9] Ralph J Hayashi T What makes a good monolignol substitute? The Science and Lore of the Plant Cell Wall Biosynthesis, Structure and Function 2006 Boca Raton, FL: BrownWalker Press 285 293

[B10] Grabber JH Hatfield RD Methyl esterification divergently affects the degradability of pectic uronosyls in nonlignified and lignified maize cell walls J Ag Food Chem 2005 53 1546 1549 10.1021/jf048799b 15740038

[B11] Grabber JH Hatfield RD Ralph J Apoplastic pH and monolignol addition rate effects on lignin formation and cell wall degradability in maize J Ag Food Chem 2003 51 4984 4989 10.1021/jf030027c 12903957

[B12] Grabber JH How do lignin composition, structure, and cross-linking affect degradability? A review of cell wall model studies Crop Sci 2005 45 820 831 10.2135/cropsci2004.0191

[B13] Doane PH Schofield P Pell AN Neutral detergent fiber disappearance and gas and volatile fatty acid production during the in vitro fermentation of six forages J Anim Sci 1997 75 3342 3352 9420010 10.2527/1997.75123342x

[B14] Getachew G Blummel M Makkar HPS Becker K In vitro gas measuring techniques for assessment of nutritional quality of feeds: a review Anim Feed Sci Technol 1998 72 261 281 10.1016/S0377-8401(97)00189-2

[B15] Weimer PJ Dien BS Springer TL Vogel KP In vitro gas production as a surrogate measure of the fermentability of cellulosic biomass to ethanol Appl Microbiol Biotechnol 2005 67 52 58 10.1007/s00253-004-1844-7 15614558

[B16] Quideau S Ralph J Facile large-scale synthesis of coniferyl, sinapyl, and *p*-coumaryl alcohol J Agric Food Chem 1992 40 1108 1110 10.1021/jf00019a003

[B17] Ralph J MacKay JJ Hatfield RD O'Malley DM Whetten RW Sederoff RR Abnormal lignin in a loblolly pine mutant Science 1997 277 235 239 10.1126/science.277.5323.235 9211851

[B18] Ralph J Kim H Peng J Lu F Arylpropane-1,3-diols in lignins from normal and CAD-deficient pines Org Lett 1999 1 323 326 10.1021/ol9906559 10905871

[B19] Kim H Ralph J Simplified preparation of coniferyl and sinapyl alcohols J Ag Food Chem 2005 55 3693 3695 10.1021/jf047787n 15853421

[B20] Hemmerle H Burger HJ Below P Schubert G Rippel R Schindler PW Paulus E Herling AW Chlorogenic acid and synthetic chlorogenic acid derivatives: Novel inhibitors of hepatic glucose-6-phosphate translocase J Med Chem 1997 40 137 145 10.1021/jm9607360 9003513

[B21] Ulibarri G Nadler W Skrydstrup T Audrain H Chiaroni A Riche C Grierson DS Construction of the bicyclic core structure of the enediyne antibiotic Esperamicin-a(1) in either enantiomeric form from (-)-quinic acid J Org Chem 1995 60 2753 2761 10.1021/jo00114a025

[B22] Ralph J Helm RF Quideau S Hatfield RD Lignin-feruloyl ester cross-links in grasses. Part 1. Incorporation of feruloyl esters into coniferyl alcohol dehydrogenation polymers J Chem Soc, Perkin Trans 1 1992 2961 2969 10.1039/p19920002961

[B23] Lu F Ralph J Facile synthesis of 4-hydroxycinnamyl *p*-coumarates J Agric Food Chem 1998 46 2911 2913 10.1021/jf980440y

[B24] Grabber JH Ralph J Hatfield RD Quideau S Kuster T Pell AN Dehydrogenation polymer-cell wall complexes as a model for lignified grass walls J Agric Food Chem 1996 44 1453 1459 10.1021/jf9502717

[B25] Grabber JH Lu F Formation of syringyl-rich lignins in maize as influenced by feruloylated xylans and *p*-coumaroylated monolignols Planta 2007 226 741 751 10.1007/s00425-007-0521-3 17457604

[B26] Hatfield RD Jung HG Ralph J Buxton DR Weimer PJ A comparison of the insoluble residues produced by the Klason lignin and acid detergent lignin procedures J Sci Food Agric 1994 65 51 58 10.1002/jsfa.2740650109

[B27] Kim H Ralph J Solution-state 2D NMR of ball-milled plant cell wall gels in DMSOd6/pyridine-d5 Org Biomol Chem 2010 8 576 591 10.1039/b916070a 20090974 PMC4070321

[B28] Weimer PJ Mertens DR Ponnampalam E Severin BF Dale BE FIBEX-treated rice straw as a feed ingredient for lactating dairy cows Anim Feed Sci Technol 2003 103 41 50 10.1016/S0377-8401(02)00282-1

[B29] Dubois M Giles KA Hamilton JK Rebers PA Smith F Colorimetric method for determination of sugars and related substances Anal Chem 1956 28 350 356 10.1021/ac60111a017

[B30] Steel RGD Torrie JH Principles and procedures of statistics 1980 2 New York: McGraw-Hill Publishing Co

[B31] SAS SAS PC Windows Version 9.1.3 2003 USA: SAS Institute Inc, Cary, NC

[B32] Ferrer MA Barcelo AR Inactivation of cell wall acidic peroxidase isoenzymes during the oxidation of coniferyl alcohol in *Lupinus* Phytochemistry 1994 36 1161 1163 10.1016/S0031-9422(00)89630-2

[B33] Huang L Colas C Ortiz de Montellano PR Oxidation of carboxylic acids by horseradish peroxidase results in prosthetic heme modification and inactiviation J Am Chem Soc 2004 126 12865 12873 10.1021/ja046455w 15469283

[B34] Huang Q Huang Q Pinto RA Griebenow K Schweitzer-Stenner R Weber WJ Jr Inactivation of horseradish peroxidase by phenoxyl radical attack J Am Chem Soc 2005 127 1431 1437 10.1021/ja045986h 15686375

[B35] Evans JJ Himmelsbach DS Incorporation of peroxidase into synthetic lignin J Agric Food Chem 1991 39 830 832 10.1021/jf00005a002

[B36] Hatfield RD Ralph J Grabber JH A potential role for sinapyl *p*-coumarate as a radical transfer mechanism in grass lignin formation Planta 2008 228 919 928 10.1007/s00425-008-0791-4 18654797

[B37] Clifford MN Chlorogenic acids and other cinnamates - nature, occurrence and dietary burden J Sci Food Agric 1999 79 362 372 10.1002/(SICI)1097-0010(19990301)79:3<362::AID-JSFA256>3.0.CO;2-D

[B38] Arrieta-Baez D Stark RE Modeling suberization with peroxidase-catalyzed polymerization of hydroxycinnamic acids: Cross-coupling and dimerization reactions Phytochemistry 2006 67 743 753 10.1016/j.phytochem.2006.01.026 16524605

[B39] Russell WR Burkitt MJ Scobbie L Chesson A Radical formation and coupling of hydroxycinnamic acids containing 1,2-dihydroxy substituents Bioorganic Chem 2003 31 206 215 10.1016/S0045-2068(03)00042-7 12818230

[B40] Guyot S Vercauteren J Cheynier V Structural determination of colourless and yellow dimers resulting from (+)-catechin coupling catalysed by grape polyphenoloxidase Phytochemistry 1996 42 1279 1288 10.1016/0031-9422(96)00127-6

[B41] Hosny M Rosazza JPN Novel oxidations of (+)-catechin by horseradish peroxidase and laccase J Ag Food Chem 2002 50 5539 5545 10.1021/jf020503j 12236676

[B42] Kusano R Tanaka T Matsuo Y Kouno I Structures of epicatechin gallate trimer and tetramer produced by enzymatic oxidation Chem Pharm Bull 2007 55 1768 1772 10.1248/cpb.55.1768 18057757

[B43] Grabber JH Hatfield RD Ralph J Zon J Amrhein N Ferulate cross-linking in cell walls isolated from maize cell suspensions Phytochemistry 1995 40 1077 1082 10.1016/0031-9422(95)00413-2

[B44] Oosterveld A Grabber JH Beldman G Ralph J Voragen AGJ Formation of ferulic acid dehydrodimers through oxidative cross-linking of sugar beet pectin Carbohydr Res 1997 300 179 181 10.1016/S0008-6215(97)00041-4

[B45] Bunzel M Ralph J Funk C Steinhart H Isolation and identification of a ferulic acid dehydrotrimer from saponified maize bran insoluble fiber Eur Food Res Technol 2003 217 128 133 10.1007/s00217-003-0709-0

[B46] Weimer PJ Russell JB Muck RE Lessons from the cow: What the ruminant animal can teach us about consolidated bioprocessing of cellulosic biomass Bioresource Tech 2009 100 5323 5331 10.1016/j.biortech.2009.04.075 19560344

[B47] Grabber JH Mertens DR Kim H Funk C Lu F Ralph J Cell wall fermentation kinetics are impacted more by lignin content and ferulate cross-linking than by lignin composition J Sci Food Agric 2009 89 122 129 10.1002/jsfa.3418

[B48] Van Soest PJ Van Amburgh ME Robertson JB Knaus WF Validation of the 2.4 times lignin factor for ultimate extent of NDF digestion, and curve peeling rate of fermentation curves into pools Cornell Nutrition Conference for Feed Manufacturers; Syracuse, New York 2005 Cornell University, Ithaca, New York 139 149

[B49] Lopez S Murison SD Travis AJ Chesson A Degradability of parenchyma and sclerenchyma cell walls isolated at different developmental stages from a newly extended maize internode Acta Bot Neerl 1993 42 165 174

[B50] Buxton DR Brasche MR Digestibility of structural carbohydrates in cool-season grass and legume forages Crop Science 1991 31 1338 1345

[B51] Ralph J Lapierre C Marita J Kim H Lu F Hatfield RD Ralph SA Chapple C Franke R Hemm MR Van Doorsselaere J Sederoff RR O'Malley DM Scott JT MacKay JJ Yahiaoui N Boudet AM Pean M Pilate G Jouanin L Boerjan W Elucidation of new structures in lignins of CAD- and COMT-deficient plants by NMR Phytochemistry 2001 57 993 1003 10.1016/S0031-9422(01)00109-1 11423146

[B52] Ralph J Schatz PF Lu F Kim H Akiyama T Nelsen SF Rokita S Quinone methides in lignification Quinone Methides 2009 Hoboken, NJ: Wiley-Blackwell

[B53] Kim H Ralph J Akiyama T Solution-state 2D NMR of ball-milled plant cell wall gels in DMSO-d6 Bioenerg Res 2008 1 56 66 10.1007/s12155-008-9004-z

[B54] Zhu N Huang TC Yu Y LaVoie EJ Yang CS Ho CT Identification of oxidation products of (-)-epigallocatechin gallate and (-)-epigallocatechin with H_2_O_2_ J Ag Food Chem 2000 48 979 981 10.1021/jf991188c 10775337

[B55] Zhu N Wang M Wei GJ Lin JK Yang CS Ho CT Identification of reaction products of (-)-epigallochatechin, (-)-epigallochatechin gallate and pyrogallol with 2,2-diphenyl-1-picrylhydrazyl radical Food Chem 2001 73 345 349 10.1016/S0308-8146(00)00308-3

[B56] Ralph SA Landucci LL Ralph J NMR Database of Lignin and Cell Wall Model Compounds http://ars.usda.gov/Services/docs.htm?docid=10429 updated at least annually since 1993.

